# NPAideS: a drug-checking study among 3-methylmethcathinone (3-MMC) users

**DOI:** 10.1186/s12954-023-00836-4

**Published:** 2023-07-28

**Authors:** Théo Willeman, Nathan Grundig, Christine Pochon, David Michels, Nicolas Charpentier, Hélène Eysseric-Guérin, Nathalie Fouilhé Sam-Lai, Françoise Stanke-Labesque, Bruno Revol

**Affiliations:** 1 Univ. Grenoble Alpes, Laboratoire de Pharmacologie, Pharmacogénétique, Toxicologie, CHU Grenoble Alpes, Grenoble, France; 2 Univ. Grenoble Alpes, Clinique de Médecine Légale, CHU Grenoble Alpes, Grenoble, France; 3 Univ. Grenoble Alpes, CEIP-Addictovigilance, CHU Grenoble Alpes, Grenoble Cedex 9, 38043 Grenoble, France; 4 CAARUD Pause Diabolo, Lyon, France; 5 AIDES, Pantin et Annemasse, Annemasse, France; 6 Laboratoire de Recherche Communautaire, Coalition PLUS, Pantin, France; 7 Univ. Grenoble Alpes, Laboratoire HP2 Inserm, U1300 Grenoble, France; 8 Institute of Sociological Research (ISR), University of Geneva, Geneva, Switzerland

**Keywords:** Chemsex, 3-methylmethcathinone, Harm reduction, Drug checking

## Abstract

**Background:**

3-methylmethcathinone (3-MMC) has been available on the European drug market for several years, but an increase in its availability seems to have occurred around 2020, associated with reports of harm and death. We aimed to analyze the composition of the supposed 3-MMC samples purchased and its concordance with the assumed composition of the drug.

**Methods:**

A prospective multicenter (*n* = 6) study was conducted between February 2021 and September 2021 in Auvergne-Rhone-Alpes, France. The inclusion criteria were: 3-MMC users over 18 years of age in contact with a community-based organization (CBO) called AIDES. Consumption was evaluated with an anonymized questionnaire and samples of 3-MMC powder were analyzed with a combination of qualitative (GC–MS) and quantitative methods (UPLC-MS/MS), to compare the assumed and real compositions of the products purchased.

**Results:**

We studied 45 samples provided by 33 users. The study population was predominantly male (91%), with a median age of 40 years, most were university graduates and regular users of 3-MMC. Intravenous drug use was reported by 15.2% of the population. Most of the users bought their 3-MMC online via the Clear Web. Drug testing was requested by 86% of the users, highlighting the need for this type of harm reduction strategy. The purity of the 3-MMC powder samples tested ranged from 21 to 98%. Other NPS drugs, such as 4-CEC (4-chloroethcathinone), 4-MMC, and 2-fluorodeschloroketamine (2-FDCK), supplied as methoxphenidine (MXP), were also detected.

**Conclusion:**

This prospective study shows that 3-MMC purity and dose vary considerably. It also describes the characteristics of 3-MMC users and their expectations of a drug-checking program. Our data suggest that drug-checking services may be useful in this population. Health associations and laboratories should work together to help increase access to such programs.

## Introduction

Synthetic cathinones are a group of stimulants chemically related to the main psychoactive substance in the khat plant (*Catha edulis*). They are sold as “legal” replacements for controlled stimulants, such as amphetamine, MDMA, and cocaine. At the end of 2021, the European Monitoring Centre for Drugs and Drug Addiction (EMCDDA) was monitoring 162 cathinones, making these drugs the second largest category of new psychoactive substances (NPS) monitored in the European Union, after synthetic cannabinoids [1]. Most cathinone use appears to be recreational, including use in high-risk settings, such as “chemsex” parties.

“Chemsex” can be defined as the use of psychoactive substances before or during planned sexual events to facilitate, enhance, prolong, and sustain the experience [2]. Chemsex is mostly practiced by men who have sex with men (MSM) [3]. “Slam” practices are defined as the intravenous injection of psychoactive substances in a “chemsex” context [2]. Worrying increases in the rates of complications (risky, unprotected, and seroconversion-associated behaviors, infections with HIV or HCV, psychiatric disorders, acute neurological symptoms) and deaths related to “chemsex” have highlighted the need for specific care and information pathways [4]. Synthetic cathinones, such as 4-methyl-N-ethylcathinone (4-MEC), 3-methylmethcathinone (3-MMC), and mephedrone (4-MMC), are often used in chemsex, with or without γ-hydroxybutyrate (GHB), and can cause fatal acute poisoning [4, 5]. 3-MMC is generally administered by insufflation, inhalation, orally or by injection, and is easy to purchase online at an affordable price [6]. 3-MMC first came to prominence in Sweden in 2012 [7], but there has been a recent marked increase in the supply of cathinone powder in Europe. In 2020, 3-MMC was the most frequently identified substance in NPS seizures in Italy [8].

Harm reduction strategies are therefore required, to reduce the risk of complications. Drug-checking services (DCS) are particularly useful as they provide users with information about the content of samples, together with advice and, in some cases, counseling or brief interventions. They constitute a valuable tool of demonstrated efficacy for public health services, as they facilitate access to the population of drug users [9, 10]. Such services are now well established for opioids, but they remain marginal for stimulants [11]. However, DCS programs for recreational drugs, such as cocaine and MDMA, led to the early detection of NPS, and the detection of illicit ketamine use in the Geneva area of Switzerland [12]. In 2016, a pilot study in Paris, France, focusing on harm reduction and drug checking in the context of chemsex, reported a 90% match between the presumed substance and the molecule actually identified [13]. Strong et al*.* called for collaborations to improve knowledge of chemsex as a practice in MSM populations for the development of harm reduction programs [14]. DCS may be a particularly useful tool for this purpose in this specific population.

The objectives of this study were to compare the assumed compositions of the products purchased with their real compositions, to assess 3-MMC consumption habits and to develop a harm reduction strategy based on drug-checking services focusing on 3-MMC. Indeed, 3-MMC was the substance for which use was most frequently reported to community-based organizations, and expectations were therefore high for this particular substance.

## Materials and methods

### Study design

**Fig. 1:**
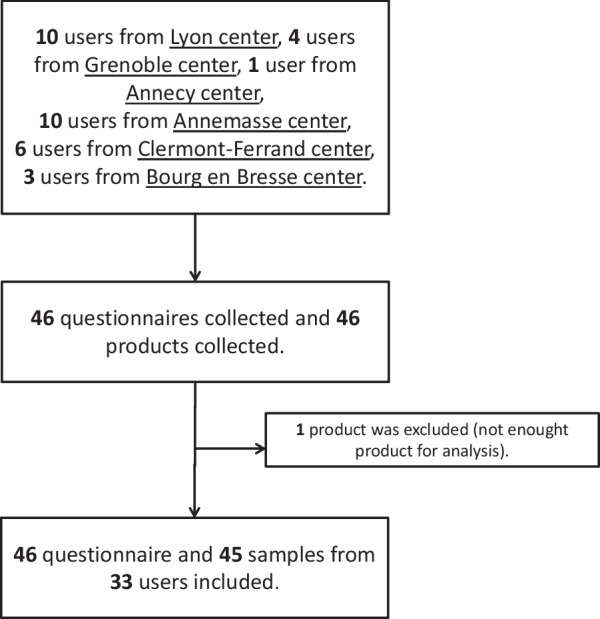
Study flowchart

This study was conducted from February 2021 to September 2021 in collaboration with several AIDES sites. It was a prospective multicenter study conducted in Auvergne-Rhone-Alpes, France, with six centers (Grenoble, Clermont-Ferrand, Lyon, Annemasse, Annecy, and Bourg-en-Bresse). Toxicological analyses were performed at the Pharmacology, Pharmacogenetics, and Toxicology Laboratory, Grenoble Alpes University Hospital, France. The inclusion criteria were: 3-MMC users over the age of 18 years in contact with the AIDES association and providing written non-opposition to the use of their data. A flowchart of the study is presented in Fig. 1. The study received ethics approval from the HDH (Health Data Hub) under number F20210126201110.

### Sample collection, evaluation of consumption, and reporting of the results

A meeting focusing on DCS and 3-MMC was organized at each center. Communication was based on flyers distributed at each center and on social networks, with the catchphrase “3-MMC, what's in your powder?”. In parallel with usual harm reduction practices, drug users were asked to provide samples (a small quantity of powder in a 1.5-mL Eppendorf tube) and to complete a questionnaire. Users were informed about the study protocol by a letter explaining the reasons for performing the study and the way in which the study would be performed. The program focused on 3-MMC users, but was also open to other NPS users. The questionnaire was anonymous and contained questions about the subject’s consumption (self-reported drug use, 3-MMC consumption frequency, routes of administration, desired effects, buying habits) and expectations of DCS (wanting to know the true composition of the products consumed, their purity, the presence of adulterating agents, and expected analysis time). The results were initially communicated individually to the participants. Once all the analyses had been completed, collective feedback meetings were organized.

### Analytical methods

#### Chemicals

Analytical standards (3-MMC, 4-MMC) and the internal standard (4-MMC-d3, IS) were purchased from LGC Standards (Luckenwalde, Germany). LC–MS-grade acetonitrile was purchased from VWR (Leuven, Belgium). Ultrapure water with a resistivity ≥ 18.0 MΩ.cm was produced with the Milli-Q Plus® system (Millipore, Molsheim, France). Other chemicals used were purchased from Carlo Erba reagents (Val-de-Reuil, France) or VWR.

#### Sample treatment

Stock solutions of 3-MMC were prepared in methanol at a concentration of 1 mg/L. Calibrators (7 levels from 0.1 to 100 ng/mL) and quality controls (QC) (2 levels: 5 and 60 ng/mL) were then prepared in water. The powder samples were photographed, weighed, and dissolved in methanol at a concentration of 1 mg/L. Samples of the 3-MMC stock solutions in methanol were then diluted in water by adding 10 µL of IS solution (1 µg/mL) to obtain a theoretical concentration of 100 ng/mL 3-MMC if the powder was 100% pure. Samples were immediately vortexed for 10 s and then centrifuged for 10 min at 2,000 × *g*. The resulting supernatants (200 µL) were transferred to integrated injection-ready micro-insert glass vials for quantitative analysis by liquid chromatography coupled to tandem mass spectrometry (LC–MS/MS). For gas chromatography coupled to mass spectrometry (GC–MS) screening, 50 µL supernatant was evaporated to dryness and reconstituted by adding 50  µL ethyl acetate.

#### Chromatography methods

##### Quantitative liquid chromatography method

Ultrahigh-performance liquid chromatography (UHPLC) was performed on an I-class Acquity system (Waters Milford, USA). Chromatographic separation was achieved with an Acquity HSS T3 column (100 mm × 2.1 mm, 2.5 µm) (Waters). Mobile phase A consisted of 5 mM ammonium formate, 0.1% formic acid, and mobile phase B consisted of 0.1% formic acid in ACN. The following gradient was used: 0–0.5 min: 13% B; 0.5–10 min: 13–50% B; 10–10.75 min: 50–95% B; 10.75–12.25 min: 95% B; 12.25–12.5 min: 95–13% B; 12.5–15 min: 13% B. Each analytical run lasted 15 min. The retention times were 2.62 min for 3-MMC and 2.58 for 4-MMC and 4-MMC-d3. The flow rate of the mobile phase was 0.4 mL/min. Oven temperature was set to 40 °C, and the injection volume was 1 µL. Analytes were quantified on a Xevo TQ-XS (Waters) tandem mass spectrometer, by positive electro-spray ionization (ESI) for 3-MMC or 4-MMC and 4-MMC d3, the internal standard (IS). Quantitative analysis was performed in multiple reaction monitoring (MRM) mode and the precursor-to-product ion transitions were *178.3* > *91.1* and *181.3* > *91.1* for 3-MMC/4-MMC and IS, respectively. Data were analyzed with MassLynx (v4.2, Waters).

The method was validated according to the Food and Drug Administration “Bioanalytical Method Validation Guidance for Industry” [15]. The linearity range was 0.1–100 ng/mL (coefficient of determination *r*^2^ of 0.997); the limit of quantification was set at 0.1 ng/mL, corresponding to 0.1% purity. Inter-day accuracies were 103.2 and 99% and inter-day precisions were 7.4% for internal quality controls of 5 and 60 ng/mL, respectively. Intraday accuracies were 100.6% and 101.6%, whereas intraday precisions were 2.8% and 4.7%, respectively (*n* = 6).

##### Qualitative gas chromatography

We distinguished between 3-MMC and 4-MMC and performed untargeted screening on an Agilent Technologies system combining a 7890D Network GC System with a 5977 network mass selective detector equipped with a high-efficiency source. Indeed, despite the close retention times of 3-MMC and 4-MMC on LC–MS/MS, this GC–MS method made it possible to distinguish between these isomers, with retention times of 8.70 and 8.87 min, respectively.

Samples were injected onto a DB-5 MS UI column (30 m-0.25 mm internal diameter; 0.25 µm film thickness) by pulsed split-less injection at an injector temperature of 250 °C. Temperature conditions were as follows: initial temperature of 70 °C for 1 min, increasing to 100 °C at a rate of 10 °C/min, then increasing to 300 °C at a rate of 20 °C/min and held at this temperature for 12 min. The flow rate of the carrier gas (helium) was maintained at 1 mL/min in constant-flow mode. The gas chromatograph interface temperature was held at 315 °C. Electron impact ionization was performed at 70 eV, with an ion source temperature of 230 °C and the collection of mass spectra from 40 to 600 m/z. Data were analyzed with MassHunter (v10.1, Agilent Technologies), including the Quantitative Analysis and Unknowns Analysis modules in particular.

## Results

### Population characteristics

The study population comprised 33 drug users, the characteristics of which are presented in Table 1. This population was mostly male (91%), had a median age of 40 years, and most of the users had a university degree and reported regular 3-MMC use. Intravenous drug use was reported by 15.2% of the population.

**Table 1:** Population characteristics

	*n*	%
Median age, years (min max)	40 (24–57)	
*Sex*		
Male	30	91
Female	2	6
Other (unspecified)	1	3
*Education*		
Graduate degree	19	
High school diploma	9	
Less than high school diploma	5	
*Work situation*		
Employed	19	
Unemployed	12	
Unknown	2	
*Lifetime self-report drug use (n* = *23)*		
Alcohol	16	69.9
Cannabis	15	65.2
Cocaine	9	39.1
Hallucinogens	11	47.8
Amphetamine	15	65.2
Others	15	65.2
Opiates	11	47.8
*3-MMC consumption frequency*		
More than 10 per month	12	
More than 10 per year	14	
Less than 10 per year	6	
Never	1	
*Routes of administration*		
Snorting	19	57.6
Injection	5	15.2
Oral	20	60.6
Rectal	4	12.1
*Desired effects*		
Sex enhancement	26	78.8
Sociability	15	45.5
Getting high	15	45.5
Stimulation	12	36.4
Perception modification	7	21.2
Anxiolysis	6	18.2
Relaxation	5	15.2
Pain release	3	9.1
Intellectual stimulation	2	6.1

### Buying patterns and expectations of drug-checking services

The patterns of 3-MMC purchase are shown in Table 2. Most users bought their 3-MMC online on the Clear Web (85.3%) with had confidence in their supplier (74.4%). Price was the most important criterion governing purchases (76.2%). The reasons for using a DCS and users’ expectations of such services are presented in Table 3. A higher frequency of drug testing was requested by 86% of the users, highlighting the need for harm reduction strategies of this type. The number of responses varied between questions because some users chose not to answer specific questions.

**Table 2:** 3-MMC buying patterns by users

	*n*	%
*Type of suppliers (n* = *33)*		
Internet	27	87.0
Street	3	6.5
Others	3	6.5
*Are you sure about the supplier reliability? (n* = *39)*		
Yes	29	74.4
*Was it your first choice from the website? (n* = *39)*		
Yes	31	79.5
*Search engine (n* = *34)*		
Clearweb	29	85.3
Darknet	5	14.7
*Who did advise you 3-MMC (n* = *45)*		
Surroundings	21	46.7
Internet website	11	24.4
None	12	26.7
*Influencing buying criteria (n* = *42)*		
Price	32	76.2
Formulation	28	66.7
Quantity	24	57.1
Purity	12	28.6
Dose	3	7.1
Packaging	3	7.1
*Price (n* = *43)*		
0 to 20 euros per gram	25	58.1
20 to 40 euros per gram	18	41.9

**Table 3:** Drug-checking services implementation expectations by users

	Full sample (*n* = 46)	%
Do you wish a more frequent drug-checking service? (*n* = 44)		
Yes	38	86.4
*Expectations for drug checking? (n* = *43)*		
True composition	35	81.4
Purity	28	65.1
Adulterant	28	65.1
Other	1	2.3
*Expected analysis time? (n* = *44)*		
2–4 days	18	40.9
1–2 weeks	20	45.5
1 month or more	6	13.6
*Why did you enrolled the study? (n* = *43)*		
Support the program	32	74.4
True composition	31	72.1
Evaluate new 3-MMC seller	12	27.9
Unexpected side effect	5	11.6
Unexpected product aspect	3	7.0
No or mild effect	2	4.7

### Toxicological analysis of the samples

**Fig. 2:**
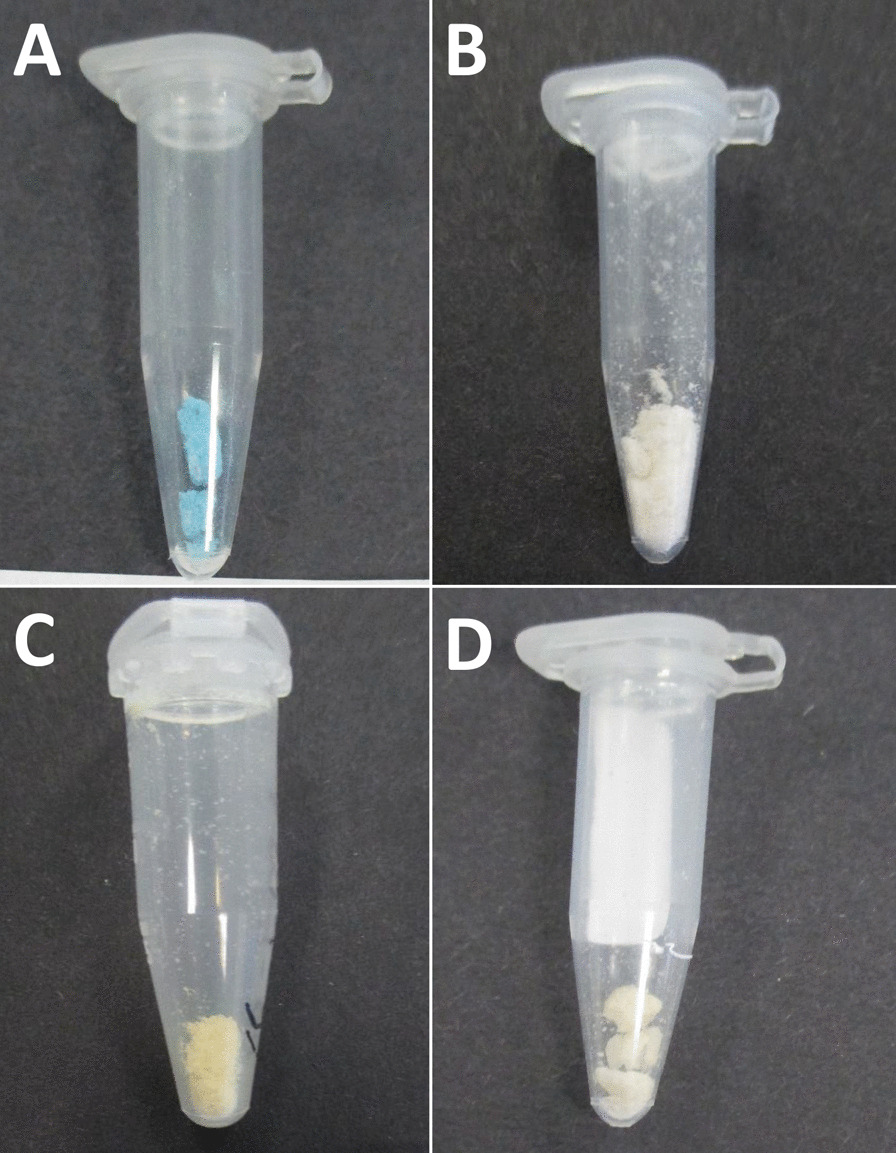
Four samples illustrating the different forms of 3-MMC

In this study, we collected and analyzed 45 samples (detailed results presented in Table 4). The purity of the 3-MMC powder samples ranged from 21 to 98%. Purity could not be determined for five samples due to insufficient amounts of sample or the lack of an analytical standard. Toxicological analyses revealed a 77% match between the presumed substance and the substance actually received by the users. One sample contained no pharmacologically active substance. Other NPS were also detected, including 4-CEC (4-chloroethcathinone), 4-MMC, and 2-fluorodeschloroketamine (2-FDCK) sold as methoxphenidine (MXP). Several adulterating agents, such as alpha-PHP and cocaine, were also detected in small amounts. However, the trace amounts detected may have been due to container reuse by the users. Several different forms of 3-MMC were identified. Most of the samples provided were in powder or crystal form, but some were provided as tablets. The different appearances of the samples are shown in Fig. 2.

**Table 4:** Samples toxicological analysis: identification, purity, and pharmacologically active adulterant identified

Expected product	Aspect	GC–MS & UPLC-MS/MS identification	Purity	Pharmacologically active adulterant identified
3-MMC	White powder	3-MMC	67%	
3-MMC	Yellowish crystal	3-MMC	81%	cocaine
3-MEC	White powder	3-MMC	21%	4-MEC
3-MMC	White crystal	3-MMC	84%	
3-MMC	White powder	3-MMC	75%	alpha-PHP
3-MMC	White powder	4-CEC	N/A	
3-MMC	Yellowish crystal	3-MMC	65%	
3-MMC	Yellowish powder	3-MMC	61.2%	
3-MMC	Blue tablet (Fig. 2A)	3-MMC	40.6%	
3-MMC	Yellowish powder	3-MMC	67.3%	
3-MMC	White powder	3-MMC	72.5%	
3-MMC	White powder	3-MMC	79.7%	
3-MMC	White powder	3-MMC	70.1%	
3-MMC	Yellowish crystal	3-MMC	IQ	
3-MMC	White powder	3-MMC	67.2%	
3-MMC	White crystal	3-MMC	69%	alpha-PHP
3-MMC	White powder	3-MMC	60.5%	
3-MMC	Yellowish powder	3-MMC	57%	
3-MMC	White powder	3-MMC	65%	
3-MMC	White powder	3-MMC	85%	
3-MMC	White powder	3-MMC	40.7%	
3-MMC	White powder (Fig. 2B)	3-MMC	70.6%	
3-MMC	White powder	3-MMC	78.2%	
3-MMC	White powder	3-MMC	84%	
3-MMC	White powder	3-MMC	57%	Caffeine
3-MMC	White powder	3-MMC	54.8%	
3-MMC	Yellowish crystal (Fig. 2D)	3-MMC	66.9%	
3-MMC	White powder	3-MMC	53%	
3-MMC	White powder	3-MMC	79.9%	
3-MMC	White powder	3-MMC	61.2%	
3-MMC	Yellowish powder	3-MMC	54.7%	
3-MMC	White powder	3-MMC	64.3%	
3-MMC	White powder	Nothing	N/A	
3-MMC	White powder	3-MMC	IQ	
3-MMC	White powder	4-MMC	89%	
3-MMC	White powder	3-MMC	77.3%	
3-MMC	Yellowish powder (Fig. 2C)	3-MMC	98%	
3-MMC	White powder	3-MMC	IQ	
3-MMC	White powder	4-MMC	82.6%	
MXP	White powder	2-FDCK	N/A	
4-MMC	White powder	3-MMC	65.2%	
4-MMC	White powder	4-MMC	63.8%	
4-MMC	White powder	4-MMC	37.9%	
4-MMC	White powder	3-MMC	55.5%	
4-MMC	White powder	4-MMC	45%	

*IQ* Insufficient quantity

*N/A* Not applicable

## Discussion

This study provided a clearer description of the 3-MMC products consumed, particularly in the “chemsex” context. The purity of the 3-MMC samples ranged from 21 to 98%, and a 77% match was found between the presumed identity of the drug and the drug actually received by users. Unexpectedly, we also detected other NPS, such as 2-FDCK. 4-CEC and 4-MMC, are also synthetic cathinones, pharmacologically similar to 3-MMC and with a similar commonly used dose. However, 2-FDCK is a potentially dangerous substance with effects very different from those of 3-MMC.

Most of the 3-MMC users studied men and the median age of the study population was 40 years. Flores Anato et al*.* identified a population of younger people and MSM who practice chemsex, with a median age of 33 years, as 3-MMC users in a pre-exposure prophylaxis (PreP) population in Canada [16]. The population of 3-MMC users in our study consisted largely of MSM practicing chemsex, although 3-MMC use seems to be expanding into the party scene [17].

The purity of the 3-MMC obtained in customs seizures has been reported to range from 45.7 to 100% [18]. In one study of 3-MMC users engaging in chemsex in France, purity ranged from 51 to 88% [13]. In 2021, the SINTES network in France analyzed 11 3-MMC samples, reporting purity values ranging from 42 to 98% [19]. We also found that purity was highly variable (21 to 98%). This variability may lead to unexpected effects, a complete absence of effect, or poisoning, because the dose consumed may vary according to the purity of the product. Knowledge about the purity of the sample would help users to adapt the quantity taken, thereby enabling them to avoid complications. Interestingly, one sample contained no psychoactive substance, but this sample was not obtained from an internet vendor. One of the 45 samples was in tablet form, all the others being in the form of powder or crystals. In the samples analyzed here, contrary to the beliefs of users, purity was not higher for crystals than for powders. Tablets have also been obtained in customs seizures, along with capsules and liquid products, albeit in smaller amounts than powders [18].

DCS can be useful to identify NPS. Indeed, new synthetic cannabinoids have been identified in this way, by gas or liquid chromatography-mass spectrometry [20]. We detected 2-fluorodeschloroketamine (2-FDCK), sold as methoxphenidine (MXP), an emerging and potentially dangerous NPS [21, 22], thereby confirming its presence in France. Drug checking for amphetamine-type stimulants is potentially useful, and personalized interventions are required for the highly diverse group of people known to use amphetamine-type stimulants [23]. In our population, the majority of who took part in chemsex, a community-based organization (CBO) was considered a good way to introduce DCS. Confidence in the vendors was high, but most users wanted DCS to determine the true composition of the products.

Several studies have reported a high level of willingness to use a formal DCS [24]. Our study confirms this finding for 3-MMC users. The main expectations that users had of these services were knowledge of the exact nature of the product, its purity and the presence of any adulterating agents. Purity determination requires quantitative analysis and, therefore, the availability of analytical standards, which may be difficult to obtain for NPS.

Laboratory analyses can take a long time from sample collection to the delivery of results, whereas on-site testing, with techniques such as thin-layer chromatography (TLC) and infrared spectroscopy [25], can yield results almost instantaneously. We found that 3-MMC were willing to wait up to two weeks for results, but alternative solutions, such as take-home drug testing with strips, might reduce that time and the potential damage due to the use of impure or contaminated products [26]. This study was a pilot study on a small group of users (*n* = 33). The upscaling of a DCS program of this type to a larger population would require good public acceptability and adaptation to the needs of users.

Liquid chromatography and gas chromatography coupled with mass spectrometry are recognized gold standard analytical method for forensic toxicology and, despite their high cost, may be suitable for use in drug checking [27]. MS is the most discriminatory drug testing technique, but it can be hard to implement in the field, due to its high cost and the need for trained staff. High-resolution mass spectrometry (HRMS) could help to identify new compounds in powders of unknown composition [28].

This study has several limitations. Samples were supplied by volunteers, via AIDES, who may not be representative of all 3-MMC users. Furthermore, sample collection was geographically limited. The toxicological analyses searched only for pharmacologically active adulterating agents, due to the choice of analytical methods. We did not check for the presence of inorganic compounds, such as salts.

In addition, following the introduction of controls on 3-MMC in the Netherlands in October 2021, there are indications that some online vendors have started to offer 3-chloromethcathinone (3-CMC) as a replacement for 3-MMC [29]. In March 2022, the European Commission also adopted measures to control 3-MMC and 3-CMC [30]. Together, these decisions could have a major impact on the availability and quality of these products.

## Conclusion

This harm reduction strategy based on DCS provided us with a more detailed description of the products used, patterns of use, and the reasons for which 3-MMC users requested drug checking. DCS may be useful in this population, which requests product testing more frequently than other groups. Collaboration between CBOs and hospital laboratories could help spread programs of this type.

## Data Availability

The datasets used and/or analyzed in this study are available from the corresponding author on reasonable request.
